# Edge Channel Transmission through a Quantum Point Contact in the Two-Dimensional Topological Insulator Cadmium Arsenide

**DOI:** 10.1021/acs.nanolett.3c01263

**Published:** 2023-06-12

**Authors:** Simon Munyan, Arman Rashidi, Alexander C. Lygo, Robert Kealhofer, Susanne Stemmer

**Affiliations:** Materials Department, University of California, Santa Barbara, California 93106-5050, United States

**Keywords:** Topological insulator, quantum point contact, quantum Hall effect, quantum interference, spin−orbit coupling

## Abstract

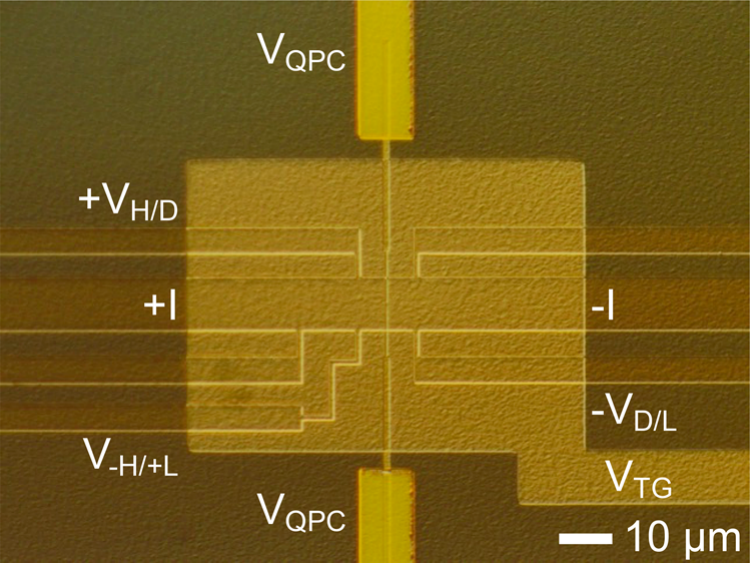

Cadmium arsenide (Cd_3_As_2_) thin films feature a two-dimensional topological insulator (2D TI) phase for certain thicknesses, which theoretically hosts a set of counterpropagating helical edge states that are characteristic of a quantum spin Hall (QSH) insulator. In devices containing electrostatically defined junctions and for magnetic fields below a critical value, chiral edge modes of the quantum Hall effect can coexist with QSH-like edge modes. In this work, we use a quantum point contact (QPC) device to characterize edge modes in the 2D TI phase of Cd_3_As_2_ and to understand how they can be controllably transmitted, which is important for use in future quantum interference devices. We investigate equilibration among both types of modes and find non-spin-selective equilibration. We also demonstrate the effect of the magnetic field on suppressing equilibration. We discuss the potential role of QSH-like modes in a transmission pathway that precludes full pinch-off.

Quantum point contacts (QPCs) allow for selective and controlled transmission of individual integer and fractional quantum Hall edge modes,^1^ opening avenues for fundamental physics studies of quantum Hall states and novel device applications. Such applications include electron optical devices, quantum Hall interferometry, and detection of non-Abelian quasi-particle statistics.^2−6^ To date, only a few investigations of QPCs have focused on topologically nontrivial materials.^7^ QPCs in two-dimensional topological insulators (2D TIs) would allow for advancing the experimental understanding of their unique edge states and create building blocks for interference experiments that would be useful for future topological quantum information systems. For example, the manipulation of counterpropagating helical edges states with QPCs has been proposed for studies of quantum entanglement, creation and detection of Majorana bound states, and studies of interactions.^7−14^

Epitaxial cadmium arsenide (Cd_3_As_2_) thin films provide a platform for realizing QPCs in a topological material. In particular, recent work has found that thin, (001)-oriented Cd_3_As_2_ films possess a nontrivial gap in their two-dimensional electronic states and are thus expected to host helical edge modes characteristic of the quantum spin Hall (QSH) state.^15^ Similar to graphene,^16−20^ both electron- and hole-like edge channels can be simultaneously present in electrostatically defined QPCs. Moreover, as schematically shown in Figure 1a, when the Fermi level is in the gap of the 2D TI, a set of counterpropagating, spin-polarized edge modes exists below a critical field (*B*_c_). We denote these edge channels as QSH-like, since they are not topologically protected by time reversal symmetry, in contrast to the helical edge modes at *B* = 0, and therefore there may be a mini-gap in the edge states.^21−23^ In a locally gated device, these QSH-like edge states can coexist with chiral edge states of the quantum Hall effect.^24^ At *B* = *B*_c_ the band order transitions to trivial (uninverted), and for *B* > *B*_c_ there are no edge states when the Fermi level is located in the gap (filling factor ν = 0).

**Figure 1:**
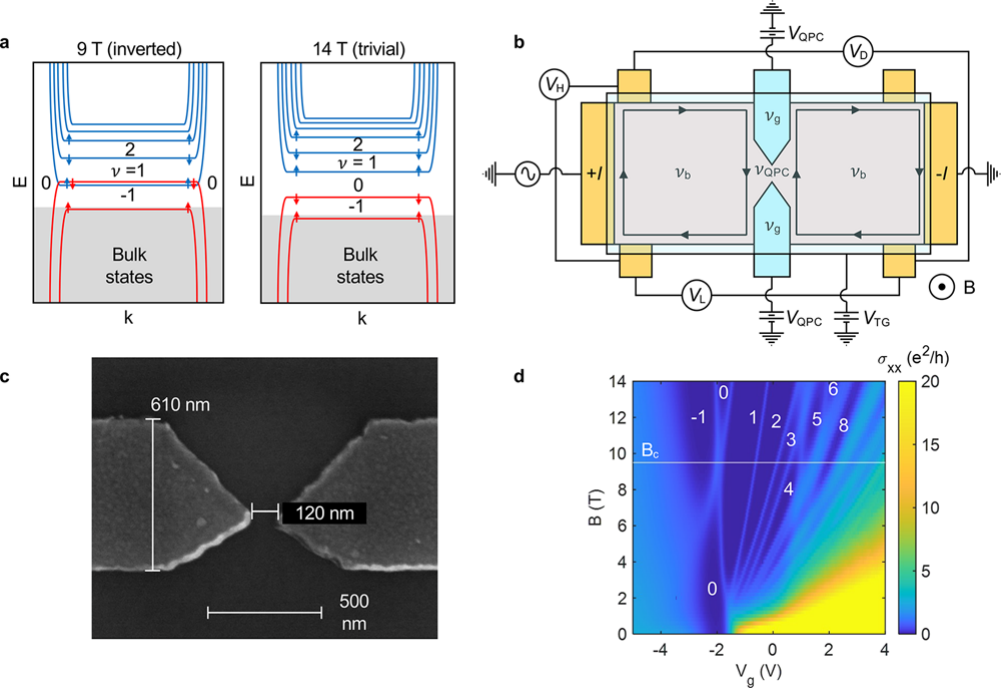
QPC device and Landau level spectrum. (a) Schematic of Landau levels and edge states of a typical 2D TI in the inverted state at *B* < *B*_c_ (9 T) and in the trivial (uninverted) regime (14 T). Gray areas mark the energy ranges where heavy hole states do not allow for higher-order hole-like Landau levels to be resolved (left region in the experimental Landau level spectrum shown in Figure 1d). (b) QPC device schematic and contact configurations. (c) Scanning electron micrograph of representative QPC split gates from a different chip fabricated using the same parameters. (d) Landau level spectrum showing longitudinal conductivity as a function of magnetic field *B* and gate voltage *V*_g_, measured on a Hall bar device. The filling factor ν is assigned based on the quantum Hall plateaus shown in Figure S2.

In this work, we report on a QPC device fabricated on a Cd_3_As_2_ thin film. We demonstrate individual transmission and pinch-off of quantum Hall edge modes in quantizing magnetic fields. We show that full pinch-off is avoided in the inverted regime below *B*_c_, which may be attributed to the QSH-like modes providing a pathway for transmission when the split gates or constriction are tuned into the topological gap. When electron-like edge modes accumulate under the split gates, we observe equilibration from mixing between modes. This mixing is suppressed by sufficiently strong magnetic fields and is less efficient at higher ν. When a hole-like edge mode is localized under the split gates, we observe equilibration with reflected electron-like modes. We find that in both cases, this equilibration is not spin-selective, in contrast to other systems.

Figure 1b shows a schematic top view of the device. Electron beam lithography (EBL) was used to pattern 10 μm Hall bar mesas from a 20-nm-thick Cd_3_As_2_ film grown by molecular beam epitaxy, as described elsewhere.^25^ Mesa isolation was performed by using argon ion milling. The etched areas were filled in with an SiO_2_ field dielectric to isolate the following layers from the substrate. Ohmic Ti–Pt–Au contacts to the mesa arms were deposited via electron beam evaporation. A blanket Al_2_O_3_ gate dielectric was deposited via a 120 °C atomic layer deposition (ALD) process. The QPC split gates were patterned using EBL, followed by thermal evaporation of a 5/30 nm Ni/Au stack. Figure 1c shows that the QPC split gate separation is 120 nm. Larger gate contacts were patterned with photolithography and metallized with electron beam evaporation (Ti/Pt/Au) to make contact with the split gates. A second ALD Al_2_O_3_ gate dielectric was deposited and selectively removed from the ohmic and gate pads using an inductively coupled plasma etch. Lastly, a global top gate (5/30 nm Ni/Au) was patterned with photolithography and metallized with thermal evaporation (see Figure S1 for a micrograph of the device). All magnetotransport measurements were taken at 2 K by using a He-4 cryostat. An alternating current of 1.35 nA at 17.777 Hz was sourced, and lock-in amplifiers were used to measure voltages.

The three voltages measured are Hall (*V*_H_), diagonal (*V*_D_), and longitudinal (*V*_L_) voltages, respectively (Figure 1b). For each, the conductance is defined as *G* = *I*/*V*, where *I* is the constant source current. The voltage applied to the split gates (*V*_QPC_) electrostatically defines the constriction and also controls the filling factor, ν_g_, under the split gates, which can be estimated from the capacitance of the top gate. The voltage applied to the global top gate (*V*_TG_) determines the filling factor in the bulk of the device (ν_b_) and hence the number of edge modes flowing into the QPC, as indicated by the Hall conductance . The global top gate is screened by the underlying split gates and has no effect on the region under the split gates or on ν_g_. The (average) filling factor in the constriction (ν_QPC_) is modulated by both the split and top gates. The diagonal conductance *G*_D_ reflects the number of edge modes transmitted through the constriction and is discussed below.

A Landau level spectrum, obtained by measuring the longitudinal and Hall resistances on a gated Hall bar from the same Cd_3_As_2_ film and converting them into the longitudinal conductivity (σ_*xx*_), provides the landscape of filling factors probed by the QPC as a function of the magnetic field and global top gate voltage (Figure 1d). All degeneracies are lifted, giving rise to even and odd integer quantum Hall plateaus (see Figure S2). The zero-energy Landau levels cross at *B*_c_ ≈ 9.5 T, marking the transition between topologically nontrivial and trivial regimes (see ref (15) for a detailed discussion). In the following, we discuss the performance of the QPC in detail at two different fields: just below the phase transition (*B* = 9 T) and above it (*B* = 14 T). We first discuss results in the accumulation regime (ν_g_ > ν_b_), followed by a discussion of transmission (ν_g_ = ν_b_), pinch-off (ν_g_ < ν_b_), and inversion (ν_g_ ≤ 0).

## Accumulation Regime

**Figure 2:**
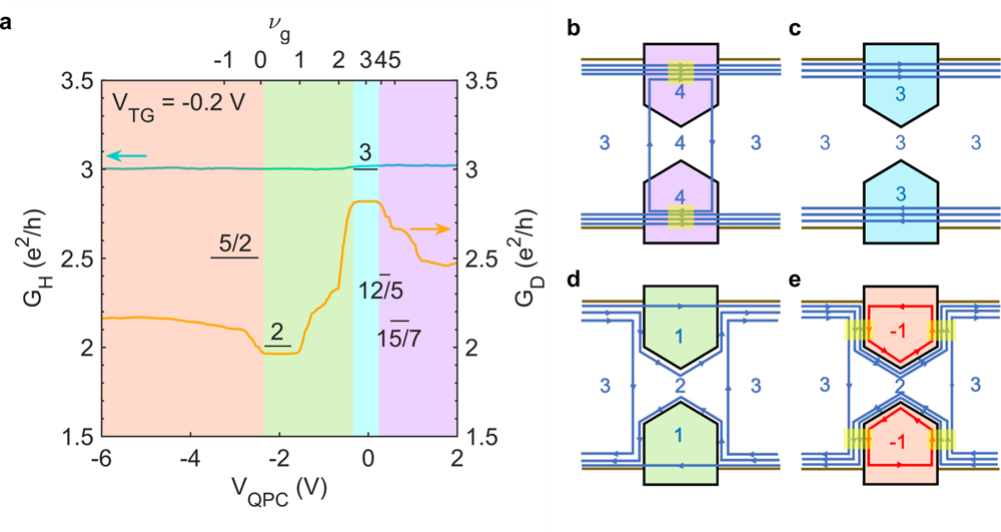
QPC operation at 9 T for ν_b_ = 3. (a) Line traces of *G*_H_ (turquoise) and *G*_D_ (orange) as a function of *V*_QPC_. The top gate voltage *V*_TG_ is set to a value such that ν_b_ = 3. Calculated values of the plateaus are shown (see text). The overlay colors indicate the following regimes: accumulation (purple), transmission (aqua), pinch-off (green), and inversion (red). (b–e) Schematics of the edge mode transport for various configurations in the QPC device. Blue modes are electron-like and red modes are hole-like. The yellow highlighted regions indicate equilibration due to edge mode mixing.

**Figure 3:**
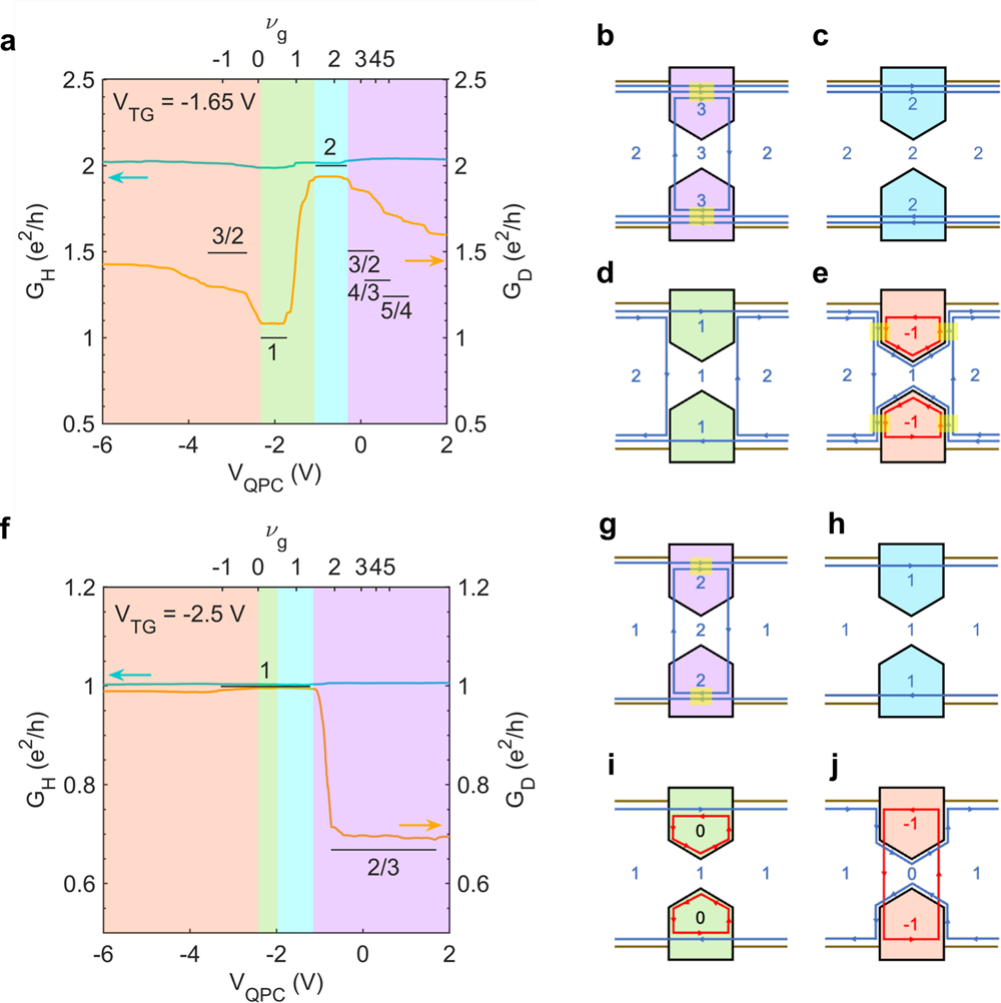
QPC operation at 9 T for ν_b_ = 2 and ν_b_ = 1. (a) Line traces of *G*_H_ (turquoise) and *G*_D_ (orange) as a function of *V*_QPC_ for ν_b_ = 2. (b–e) Schematics of edge mode transport for ν_b_ = 2. (f) Line traces of *G*_H_ (turquoise) and *G*_D_ (orange) as a function of *V*_QPC_ for ν_b_ = 1. (h–j) Schematics of edge mode transport for ν_b_ = 1. The overlay colors indicate the following regimes: accumulation (purple), transmission (aqua), pinch-off (green), and inversion (red). The yellow highlighted regions indicate equilibration due to edge mode mixing.

We begin by studying the QPCs at 9 T. In Figure 2a, *V*_TG_ is held constant, tuning the bulk to ν_b_ = 3 (*G*_H_ = 3*e*^2^/*h*), while *V*_QPC_ is modulated. In the *accumulation* regime (purple highlighted region), *V*_QPC_ is tuned such that ν_*g*_ > ν_*b*_ and additional electron-like modes form under the split gates, as schematically shown in Figure 2b. These localized modes can short-circuit the constriction for  and equilibrate opposite edges of the device, decreasing the conductance. This equilibration gives rise to the series of steps in *G*_D_ seen in Figure 2a as ν_g_ changes. This scenario is similar to an *n*-*n*′-*n* junction, for which the conductance is given by^17^
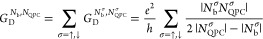
1where σ denotes the spin polarization of the associated Landau level and (*N*_b_^σ^,*N*_QPC_^σ^) give the number of equilibrating edge modes. A complete set of values of *G*_D_ calculated from eq 1 for ν_b_ = 3 to ν_b_ = 1 are shown in Table S1. For ν_b_ = 3 at 9 T (Figure 2a), *G*_D_ exhibits steps at ν_g_ = 4 and ν_g_= 5, indicative of equilibration between the three channels from the bulk with the additional channels under the split gates (see, e.g., Figure 2b for ν_g_ = 4). The calculated values for *G*_D_^3,4^=12/5 and *G*_D_^3,5^=15/7 are marked in Figure 2a for these steps, which are far below the actual values. A series of steps also occurs in the accumulation regime for ν_*b*_ = 2 (Figure 3a, purple highlighted region) when ν_g_ > 2. The values of these steps are also higher than the calculated values.

The results for ν_b_ = 3 and ν_b_ = 2 thus suggest an incomplete degree of equilibration between the edge modes, which may be explained through a picture of the quantum Hall effect where the edge modes correspond to conducting, compressible strips separated by insulating, incompressible strips.^26^ In this model, mixing between edge modes involves tunneling through the incompressible strips. Therefore, we expect mixing to be less efficient for higher-order edge modes, because charge must tunnel through multiple incompressible strips.^27,28^ This picture is confirmed by what is seen for ν_b_ = 1 (Figure 3f), where a sharp drop in the conductance from *e*^2^/*h* to ∼0.7*e*^2^/*h* closely matches the expected value of *G*_D_^1,2^ = (2/3)*e*^2^/*h*. Equilibration is thus much more complete for ν_b_ = 1, owing to the fewer incompressible strips. Furthermore, this result demonstrates that mixing between electron-like channels is not spin-selective, since the two edge modes involved have opposite spin polarizations.

**Figure 4:**
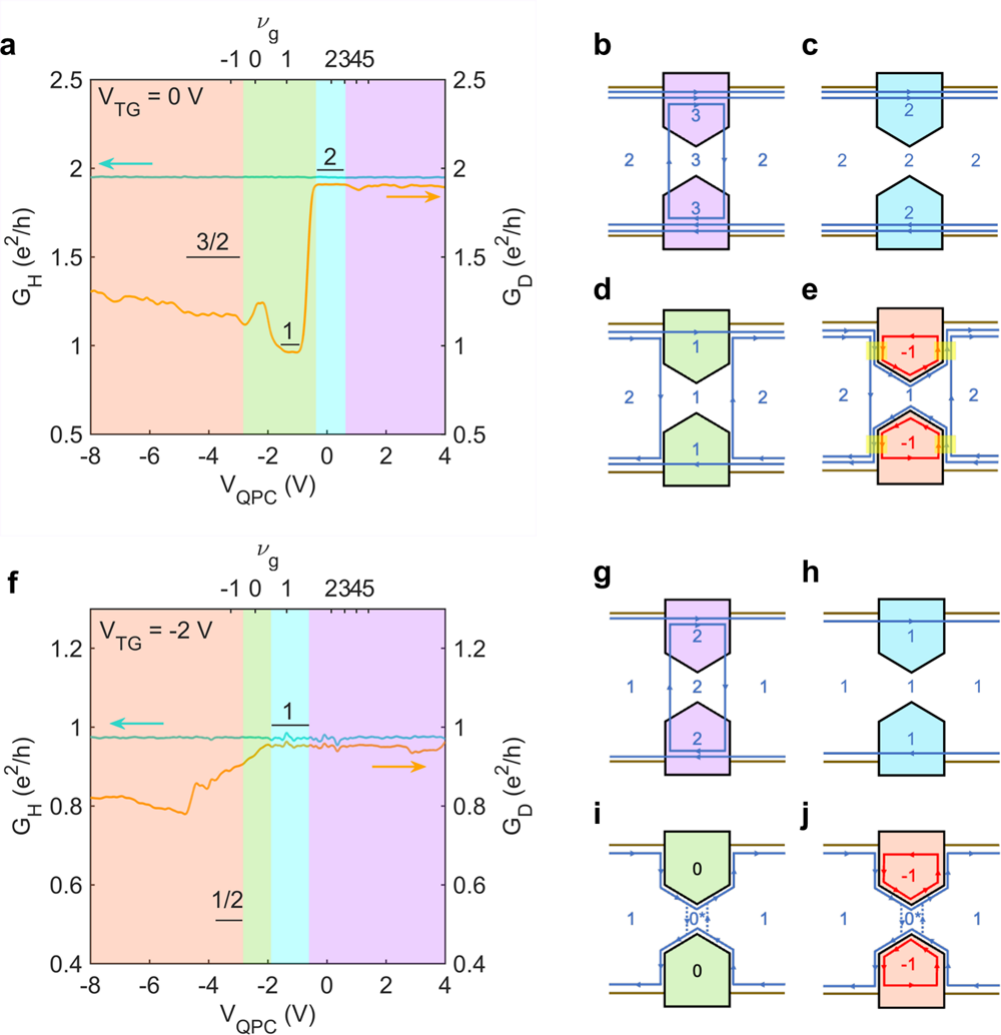
QPC operation at 14 T for ν_b_ = 2 and ν_b_ = 1 **(**a) Line traces of *G*_H_ (turquoise) and *G*_D_ (orange) as a function of *V*_QPC_ for ν_b_ = 2. (b–e) Schematics of edge mode transport for ν_b_ = 2. (f) Line traces of *G*_H_ (turquoise) and *G*_D_ (orange) as a function of *V*_QPC_ for ν_b_ = 1. (h–j) corresponding schematics of edge mode transport. The overlay colors indicate the following regimes: accumulation (purple), transmission (aqua), pinch-off (green), and inversion (red). The yellow highlighted regions indicate equilibration due to edge mode mixing.

In contrast to the data at 9 T, there are no signs of equilibration under accumulation at 14 T (Figure 4), as *G*_D_ remains constant for both ν_b_ = 2 (Figure 4a) and ν_b_ = 1 (Figure 4f). This suggests that mixing among the electron-like edge states is reduced at 14 T. In the model discussed previously, higher magnetic fields increase the width of the incompressible strips, making tunneling between all edge modes less efficient.^26^ Evidently, at 14 T the strips are wide enough to completely quench mixing.

## Transmission and Pinch-Off Regimes

As *V*_QPC_ is decreased, the device transitions from accumulation to transmission where . For ν_b_ = ν_g_ = 3 (the aqua region in Figure 2a), *G*_D_ plateaus at nearly 3*e*^2^/*h*. This plateau indicates transmission of the three edge modes underneath the split gates, as schematically shown in Figure 2c. The degree of quantization is poor, likely due to residual bulk transport at higher filling factors. Accordingly, quantization is much better at ν_b_ = ν_g_ = 2 and , see Figure 3a and f.

Upon decreasing *V*_QPC_ further to ν_g_ < ν_b_, pinch-off is expected. For ν_b_ = 3 (green region in Figure 2a), the pinch-off of one edge channel is seen, which results in a sharp decrease to a plateau at *G*_D_ = 2*e*^2^/*h*. This plateau occurs for ν_g_ = 1, meaning that the outermost edge mode passes under the split gates, while the other edge mode passes through the constriction, as illustrated in Figure 2d. A similar situation occurs for ν_b_ = 2 (Figure 3a), where *G*_D_ drops from 2*e*^2^/*h* to *e*^2^/*h*. At ν_b_ = 1 (Figure 3f), however, there is no pinch-off at any value of ν_g_, even when ν_g_ reaches −1. A possible reason is illustrated in Figure 3j and will be discussed below. In contrast, for ν_b_ = 1 at 14 T (Figure 4f), full transmission occurs at ν_g_ = 1, followed by a gradual decrease in *G*_D_ for ν_g_ ≤ 0 that indicates partial pinch-off, in contrast to the 9 T data.

## Inversion Regime

In the inversion regime (ν_g_ ≤ 0, red highlighted regions in Figures 2–4), a hole-like mode forms under the split gates, which can mix with incoming electron-like modes, causing equilibration. In this regime, only mixing with reflected electron-like modes has an effect on *G*_D_, contributing a fraction of *e*^2^/*h*, whereas transmitted modes will contribute a full *e*^2^/*h*. For example, for ν_b_ = 3 (Figure 2a), the increase of *G*_D_ for ν_g_ = −1 corresponds to equilibration between the back-reflected channel and the localized hole-like channel, as illustrated in Figure 2e. Notably, this mixing indicates a lack of spin-selectivity, as the equilibrating channels have opposite spin polarization, which is a departure from findings in other material systems.^20,27,28^ In general, spin-flip transitions may be facilitated by spin-orbit interaction,^24,29−31^ which is strong in Cd_3_As_2_.^32,33^ For this scenario, the expected quantitative values of *G*_D_ under inversion for our device geometry have been determined in prior work^20^ to be
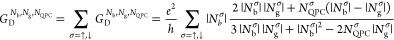
2where σ denotes the spin polarization of the associated Landau level and (*N*_*b*_^σ^, *N*_g_^σ^, *N*_QPC_^σ^) give the number of equilibrating edge modes. The expected values of *G*_D_ calculated from eq 2 are shown in Table S2 for both spin-selective and non-spin-selective mixing. For ν_b_ = 3 and ν_g_ = −1 (Figure 2a) non-spin-selective equilibration occurs between the back-reflected channel and the localized hole-like channel, as shown in Figure 2e. Such a scenario results in *G*_D_^1,–1,0^ = (1/2)*e*^2^/*h* from mixing plus an additional *G*_D_^2,0,2^ = 2*e*^2^/*h* from the two transmitted modes, giving a total *G*_D_ = (5/2)*e*^2^/*h*. Notably, the actual value of *G*_D_ is significantly less than the calculated (5/2)*e*^2^/*h*, which may again be attributed to limited tunneling through multiple incompressible strips, similar to what is seen in the accumulation regime at 9 T. Equilibration is more complete for ν_b_ = 2 (Figure 3a), where *G*_D_ is closer to the theoretical value of *G*_*D*_^2,–1,1^ = *G*_*D*_^1,–1,0^ + *G*_*D*_^1,0,1^ = 1/2 + 1 = (3/2)*e*^2^/*h*.

In contrast to the previous cases, no signs of pinch-off are observed under inversion for ν_*b*_ = 1 (Figure 3f). A possible scenario that would explain why one edge channel is always transmitted for ν_b_ = 1 is that the constriction remains at ν_QPC_ = 1. An alternative explanation invokes an edge mode picture that is unique to a topological insulator. When ν_g_ = 0 and *B* < *B*_c_, QSH-like edge modes should form under the split gates. As illustrated in Figure 3i, the QSH-like electron mode can connect with the mode from the bulk, allowing for transmission under the split gate. As the split gates are further reduced to ν_g_ = −1, the constriction changes to ν_QPC_ = 0, and again the electron-like QSH edge mode allows transmission of the mode from the bulk (see Figure 3j). The crossing of the electron- and hole-like QSH modes as shown in Figure 3j occurs to satisfy the differences in filling factors between these various regions and the fact that edge modes cannot terminate. This crossing is facilitated by the very close spatial proximity of the two edge modes. At 9 T, the bands are nearly uninverted, and the momenta of the edge modes within the remnant nontrivial gap are approximately equal (see Figure 1a). Therefore, the modes should nearly coincide along any boundary and permit crossing. Additional compelling evidence for an interpretation that involves the QSH-like modes for the behavior at 9 T can be obtained from a comparison with the 14 T data. At 14 T, no QSH-like modes exist for ν_g_ = 0 due to the uninverted band structure. Accordingly, for ν_b_ = 1 at 14 T (Figure 4f), the onset of pinch-off of the edge mode by the QPC is seen for ν_g_ ≤ 0 by the fact that *G*_D_ falls below *e*^2^/*h*, unlike what is seen at 9 T. This situation is schematically shown in Figure 4j. Comparison of Figure 3j and Figure 4j illustrates how, for ν_QPC_ = 0, the differences in the topological states may become evident in inversion.

To briefly summarize, we studied the operation of a QPC fabricated on a (001)-oriented 2D TI Cd_3_As_2_ thin film. We demonstrated the pinch-off of integer quantum Hall edge modes. We found that full pinch-off is avoided when the device is in the inverted (topological) regime, which may be facilitated by QSH-like edge modes providing a pathway for transmission. Equilibration between edge modes is suppressed for higher filling factors and by a magnetic field, owing to the reduced tunneling probability across multiple incompressible strips that widen with increasing field. We observe non-spin-selective equilibration among hole-like and electron-like channels, likely due to the effects of spin-orbit coupling. Future work on these QPCs will focus on an improved understanding of the helical edge modes in the quantum spin Hall state at zero field, which may be achieved in QPCs with etch-defined constrictions.^7^ Furthermore, a clearer understanding of equilibration and coupling between QH and QSH-like edge modes is needed, which may be achieved in a bipolar junction device with local gating. Both of these elements are essential for optimizing future quantum point contacts and interference devices in 2DTIs.

## Data Availability

The data that support the findings of this study are available in the article and its Supporting Information. Raw data can be obtained from the corresponding authors upon request.

## Supporting Information Available

Micrograph of the device, Hall conductivity data from a Hall bar device, calculated diagonal conductances, complete set of diagonal, Hall and longitudinal conductances at 9 T from the QPC device as a function of *V*_TG_ and *V*_QPC_ (PDF)
