# Supplementary material for: Edge Channel Transmission through a Quantum Point Contact in the Two-Dimensional Topological Insulator Cadmium Arsenide
Source: Nano Lett. 2023 Jun 12;23(12):5648–53. doi: 10.1021/acs.nanolett.3c01263 (PMC10311529; doi:10.1021/acs.nanolett.3c01263)
Supplement: Supplementary file 1 — nl3c01263_si_001.pdf [file nl3c01263_si_001.pdf]

Supplementary Information

Edge channel transmission through a quantum point contact in the two-dimensional topological insulator cadmium arsenide

Simon Munyan, Arman Rashidi, Alexander C. Lygo, Robert Kealhofer and Susanne Stemmer

Materials Department, University of California, Santa Barbara, CA 93106-5050, USA.

Device Layout

The wide arm of the Cd_3As_2 mesa (10 µm wide) is used for source and drain currents. Narrow arms of the mesa are used to measure voltage. A voltage is applied to the wide vertical gold leads to modulate the QPC split gates. A dielectric layer (20 nm Al_2O_3) separates the split gates from the global top gate. The top gate covers the entire device area and modulates the overall carrier density.

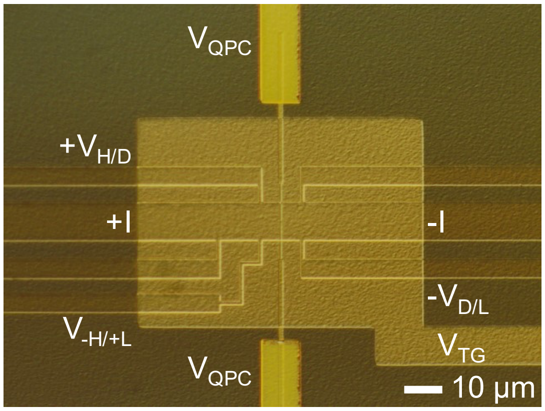

Fig. S1: Quantum point contact device.

Hall conductivity

The longitudinal conductivity (σ_{xx}), shown in the main text, and the Hall conductivity (Fig. S2) of a Hall bar of the same Cd_3As_2 film were calculated from the resistivities measured using a four-probe technique following tensor inversion:

$$\sigma_{xx} = \frac{\rho_{xx}}{\rho_{xy}^2+\rho_{xx}^2} \tag{S1}$$

$$\sigma_{xy} = \frac{-\rho_{xy}}{\rho_{xy}^2+\rho_{xx}^2} \tag{S2}$$

where ρ_{xy} is the Hall resistivity and ρ_{xx} is the longitudinal resistivity. In the 9 T trace (Fig. S2b), a kink at $\sigma_{xy} = 0$ marks the $\nu = 0$ plateau. At 9 T this plateau is not very pronounced, due to the proximity to the critical field B_c where the zero Landau levels cross. The plateaus at $\nu = 5$ and 6 are obscured by an additional subband, which are seen as a pair of nearly vertical features in the Landau level spectrum in the main text (see ref. [1] for a detailed discussion). In the 14 T trace (Fig. S2c), the plateaus at $\nu = 3$ and 4 are obscured by the same additional subband. At both fields, for gate voltages V_g below the $\nu = -1$ plateau, the Hall conductivity gradually approaches zero, which is due to an increasing carrier density coming from the bulk valence band. This regime is seen as a region of higher longitudinal conductivity for $V_g \lesssim -3\ V$.

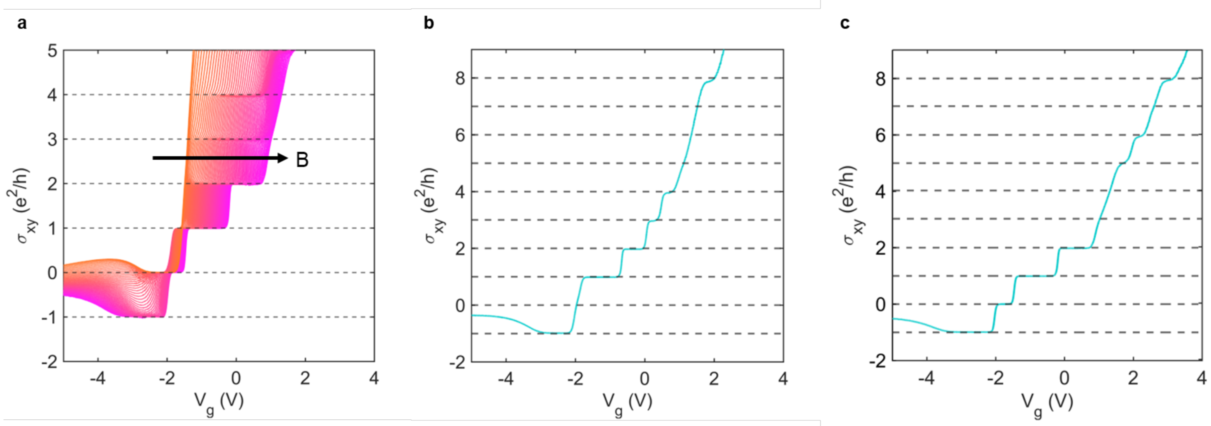

Fig. S2: **Hall conductivity as a function of gate voltage.** (a) 0 – 14 T (b) 9 T and (c) 14 T. Dashed lines mark the quantum Hall plateaus with integer filling factors ν.

Calculated diagonal conductances in accumulation

The diagonal conductance G_{D} for a given number of modes in the bulk (ν_{b}) and crossing the constriction (ν_{QPC}) were calculated using Eq. (1) in the main text and the results are shown in Table SI. Conductances for both spin-selective equilibration ("spin") and non-selective ("no spin") are considered. It should be noted that although the modes which accumulate under the split gates are responsible for equilibration, they can only do so if they cross the constriction, forming a short-circuit across the device (see e.g., Fig. 2b in the main text). Therefore, the conductances depend on the filling factor in the constriction (ν_{QPC}) rather than the filling factor under the split gates (ν_{g}). This holds only in the accumulation regime, for which $\nu_{\mathrm{g}} \geq \nu_{\mathrm{QPC}} \geq \nu_{\mathrm{b}}$.

Table SI: Calculated diagonal conductances due to equilibration in the accumulation regime.

ν_b	ν_{QPC}	G_D (e^2/h) (spin)	G_D (e^2/h) (no spin)
1	2	1	2/3 ≈ 0.67
	3	2/3 ≈ 0.67	3/5 = 0.6
	4	2/3 ≈ 0.67	4/7 ≈ 0.57
2	3	5/3 ≈ 1.67	3/2 = 1.5
	4	4/3 ≈ 1.33	4/3 ≈ 1.33
	5	19/15 ≈ 1.27	5/4 = 1.25
3	4	8/3 ≈ 2.67	12/5 = 2.4
	5	13/6 ≈ 2.17	15/7 ≈ 2.14

Calculated diagonal conductances in inversion

The diagonal conductance G_{D} in inversion for a given filling factor in the bulk (ν_{b}), under the split gates (ν_{g}), and in the constriction (ν_{QPC}) were calculated using Eq. (2) in the main text and results are shown in Table SII. Conductances for spin-selective equilibration ("spin") and non-selective equilibration ("no spin") are considered. As discussed in the main text, equilibration was found to not be spin-selective.

Table SII: Calculated diagonal conductances due to equilibration in the inversion regime.

ν_b	ν_g	ν_{QPC}	$G_D(e^2/h$ (spin)	G_D $(e^2/h$ (no spin)
1	0	0	1	1
		-1	0	1/3 ≈ 0.33
	-1	0	1	1
		-1	0	1/3 ≈ 0.33
2	0	1	3/2 = 1.5	3/2 = 1.5
		0	4/3 ≈ 1.33	4/3 ≈ 1.33
		-1	1/3 ≈ 0.33	1/2 = 0.5
	-1	1	3/2 = 1.5	3/2 = 1.5
		0	4/3 ≈ 1.33	4/3 ≈ 1.33
		-1	1/3 ≈ 0.33	1/2 = 0.5
3	0	2	2	5/2 = 2.5
		1	3/2 = 1.5	9/5 = 1.8
		0	4/3 ≈ 1.33	3/2 = 1.5
		-1	1/3 ≈ 0.33	3/5 = 0.6
	-1	2	2	5/2 = 2.5
		1	3/2 = 1.5	9/5 = 1.8
		0	4/3 ≈ 1.33	3/2 = 1.5
		-1	1/3 ≈ 0.33	3/5 = 0.6

Complete conductance data at 9 T

Figure S3 shows the complete conductance data at 9 T. The Hall conductance changes only with V_{TG} as expected, since the top gate alone modulated the bulk filling factor ν_{b}. The colored stripes are labeled according to the value of ν_{b} and are separated by solid lines. The dashed lines mark where the line traces of G_{H} and G_{D} were taken for Figs. 2 and 3 in the main text. The longitudinal conductance (G_{L}) features regions of high conductance (dark red), which correspond to the scenario of full transmission of all edge modes from the bulk. This can be verified in the expression for the longitudinal conductance derived using the Landauer-Büttiker approach [2]:

$$G_L = \frac{e^2}{h}\left(\frac{\nu_{min}\nu_{wide}}{\nu_{wide}-\nu_{min}}\right) \tag{S3}$$

where ν_{wide} is the bulk filling factor (equivalent to ν_{b}) and ν_{min} is the filling factor in the QPC constriction. In our case, modes may be transmitted by passing under the split gates or through the constriction. Therefore, the equation may be modified to read:

$$G_L = \frac{e^2}{h}\left(\frac{\min(\nu_g,\nu_{QPC})\nu_b}{\nu_b-\min(\nu_g,\nu_{QPC})}\right) \tag{S4}$$

Applying this equation to Fig. S3c, we see that the islands of high conductance correspond to the case of full transmission, where $\nu_{\mathrm{b}} = \min\,(\nu_g, \nu_{QPC})$ and G_{L} is expected to diverge. From this relation, the values of ν_{QPC} cited in the main text are inferred from the regions of high conductance where $\nu_{\mathrm{g}} < \nu_{\mathrm{b}}$, which necessarily means that $\nu_{\mathrm{QPC}} = \nu_{\mathrm{b}}$.

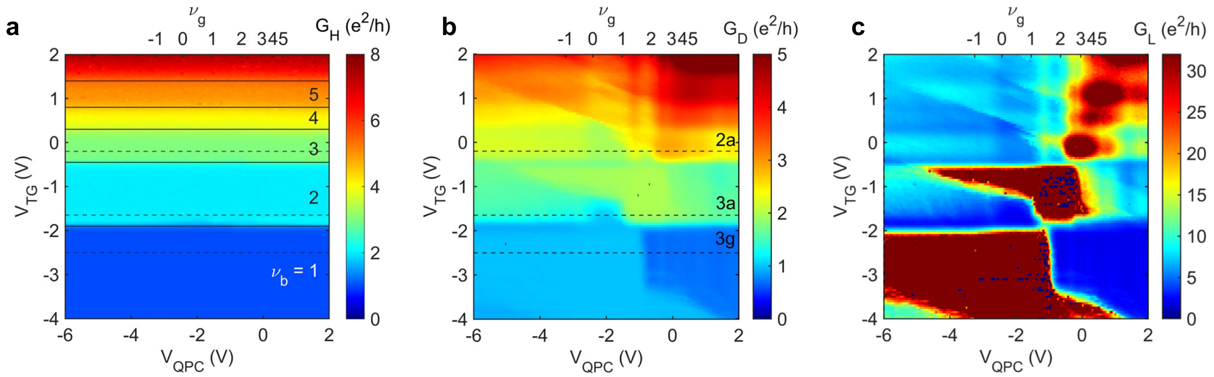

Fig. S3: **Conductance color maps at 9 T.** (a) Hall conductance G_{H}, (b) diagonal conductance G_{D}, and (c) longitudinal conductance G_{L} as a function of top gate voltage V_{TG} and split gate voltage V_{QPC}.

References

[1] A. C. Lygo, B. Guo, A. Rashidi, V. Huang, P. Cuadros-Romero, and S. Stemmer, *Two-dimensional topological insulator state in cadmium arsenide thin films*, Phys. Rev. Lett. **130**, 046201 (2023).

[2] H. Van Houten, C. W. J. Beenakker, P. H. M. Vanloosdrecht, T. J. Thornton, H. Ahmed, M. Pepper, C. T. Foxon, and J. J. Harris, *Four-terminal magnetoresistance of a two-dimensional electron-gas constriction in the ballistic regime*, Phys. Rev. B **37**, 8534-8536 (1988).
